# A Novel PCB Surface Defect Detection Method Based on the GBE-YOLOv8 Model

**DOI:** 10.3390/mi17030339

**Published:** 2026-03-10

**Authors:** Chao Gao, Xin Zhang, Mengting Bai, Xiaoqin Lian, Shichao Chen

**Affiliations:** 1 School of Computer and Artificial Intelligence, Beijing Technology and Business University, Beijing 102488, China; gaochao9158@btbu.edu.cn (C.G.); 2531062209@st.btbu.edu.cn (X.Z.); 2330602083@st.btbu.edu.cn (M.B.); 2 State Key Laboratory of Multimodal Artificial Intelligence Systems, Institute of Automation, Chinese Academy of Sciences, Beijing 100190, China

**Keywords:** PCB, defect detection, deep learning, YOLOv8n

## Abstract

In the field of printed circuit board (PCB) manufacturing, surface defect detection serves as a critical process in the production line, directly impacting the quality and safety of subsequent electronic products. However, accurately detecting tiny surface defects in real time remains a significant challenge given the complex layouts of PCBs. To address this issue, this study proposes a novel Ghost-BiFPN-Efficient-YOLOv8 (GBE-YOLOv8) model architecture for PCB defect detection based on an improved YOLOv8n. The backbone network of the model employs lightweight Ghost Conv to partially replace regular convolutions, thereby reducing computational complexity and parameter count. The neck network incorporates a multi-stage feature fusion module named G-C2f and a dynamic weighting module named BiFPN-Concat to enhance the model’s ability to characterize PCB defects. The model’s head network employs an Efficient Head that combines mixed depthwise convolution and partial convolution, further optimizing detection accuracy and computational efficiency. Simultaneously, a comprehensive evaluation of model performance was conducted using publicly available datasets. And the working mechanisms of each improved method were analyzed through class activation heatmaps to further enhance the interpretability of the model. Experimental results demonstrate that compared to the baseline model and several other state-of-the-art object detection algorithms, the proposed method exhibits significant advantages across various evaluation metrics, and its mAP@0.5, mAP@0.5:0.95, parameters, GFLOPs and FPS achieve 98.9%, 61.4%, 2.6 M, 7.5 and 252, respectively. Furthermore, each optimization method achieves the expected design purpose, and the combined application of all optimization methods enables the model to strike an optimal balance between detection accuracy and computational complexity. Consequently, this research can provide a reliable technical solution for high-precision real-time detection of surface defects on PCBs in industrial production lines.

## 1. Introduction

In the printed circuit board (PCB) quality control systems, defect detection plays a decisive role. Currently, computer vision technology has developed multi-level detection methods encompassing traditional image processing techniques such as threshold segmentation and edge detection, as well as machine learning and deep learning algorithms. These technological solutions are rapidly replacing manual inspection, enabling the intelligent upgrade of quality detection. Although automatic optical inspection (AOI) technology has been widely adopted for detecting surface defects on PCBs, the trend toward PCB integration and miniaturization presents the following challenges for machine vision-based PCB defect detection methods.

(1) The intricate physical structure of PCBs gives rise to diverse types and shapes of surface defects. These defects typically exhibit small sizes, similar characteristics, and complex backgrounds. Consequently, detection algorithms must possess robust multi-scale feature extraction capabilities while effectively distinguishing subtle differences among various defects against complex wiring backgrounds. Therefore, significantly improving the detection accuracy of small targets represents a major challenge for PCB defect detection algorithms.
(2) The production process of PCB assembly lines demands real-time defect detection. Reducing model complexity can enhance detection speed, but this often comes at the cost of some detection accuracy. Moreover, low-complexity models are advantageous for deployment on resource-constrained edge computing terminals. Therefore, effectively balancing detection accuracy and model complexity represents another challenge for PCB defect detection algorithms.

In recent years, object detection algorithms integrating machine vision and deep learning have demonstrated powerful self-learning capabilities, leading to their widespread application in PCB defect detection. Based on the mechanism for generating candidate regions, deep learning-based PCB defect detection algorithms primarily fall into two object detection paradigms: two-stage and one-stage [1]. The representative algorithms of the two-stage object detection paradigm are the R-CNN series [2,3]. Li et al. [4] employed the VGG16 [5] as the feature extraction network for Faster R-CNN and integrated RGB data augmentation techniques to achieve PCB defect detection. Hu et al. [6] further adopted ResNet50 [7] as the feature extraction network for Faster R-CNN, integrating it with the Generative Adversarial Region Proposal Network (GARPN) [8] and ShuffleNetV2 [9]. This approach enhanced the accuracy of PCB defect detection while achieving model lightweighting. Additionally, Li et al. [10] proposed a detector based on the Feature Pyramid Network (FPN). By integrating Faster R-CNN and FPN as its foundational architecture and employing RoIAlign [11] technology, the model significantly enhanced its detection performance for tiny defects on PCBs. The two-stage object detection paradigm suffers from slower inference speeds due to its higher model complexity. Representative algorithms of the one-stage object detection paradigm include SSD [12] and the YOLO series [13]. Shi et al. [14] proposed an enhanced SSD network that effectively boosts the model’s detection capability for tiny PCB defects by propagating deep features to shallow layers through a semantic mapping module. Chen et al. [15] introduced a Transformer-YOLO detection model employing the Swin Transformer as the feature extraction network, significantly improving the model’s accuracy in detecting PCB defects. Ling et al. [16] proposed an improved YOLOv8 detection model that incorporates lightweight ghost convolutions to reduce computational overhead, effectively accelerating the model’s detection speed for PCB defects. Compared to the two-stage object detection paradigm, the one-stage approach typically features lower model complexity, better aligning with the real-time requirements of PCB defect detection. However, under the complex physical structures of PCBs, accurately detecting various tiny defects in real time remains an unsolved challenge. In the context of this study, tiny defects are defined as those accounting for less than 0.25% of the total image size.

To address the aforementioned issues, considering YOLOv8n’s balanced performance in detection accuracy and speed, this study selects YOLOv8n as the baseline model. We optimize its backbone network, neck network, and head network to enhance the model’s feature extraction, feature fusion, and object detection capabilities, respectively. The main contributions of this study are as follows.

(1) This study proposes a novel Ghost-BiFPN-Efficient-YOLOv8 (GBE-YOLOv8) model architecture based on an improved YOLOv8n. Compared with the baseline YOLOv8n, the mAP@0.5, mAP@0.5:0.95 and FPS of GBE-YOLOv8 increased by 3.1%, 11.7% and 8.2%, respectively, while the parameters and GFLOPs of GBE-YOLOv8 decreased by 13.3% and 7.4%, respectively.
(2) The lightweight Ghost Conv module is introduced into the backbone network of the model to partially replace regular convolutions, thereby reducing computational complexity and parameter count. This enhancement accelerates the model’s detection speed for PCB defects.
(3) The model incorporates a multi-stage feature fusion module named G-C2f and a dynamic weighting module named BiFPN-Concat within its neck network. These components enhance the model’s ability to characterize PCB defects, thereby improving detection accuracy.
(4) An Efficient Head combining mixed depthwise convolution and partial convolution is designed in the model’s head network to further optimize detection accuracy and computational efficiency.

The structure of this article is organized as follows. Section 2 comprehensively reviews the current state of research on PCB defect detection methods. Section 3 details the network architecture and improvement methods of the GBE-YOLOv8 model. Section 4 validates the proposed algorithm using public datasets and compares its performance with other mainstream advanced detection models. Section 5 presents the research conclusions and outlines future research directions.

## 2. Related Work

### 2.1. Vision-Based Methods for PCB Surface Defect Detection

Early PCB surface defect detection relied entirely on manual visual inspection, which is still employed by many companies today. However, as demands for production quality and efficiency have increased, the inefficiency and high miss rate of manual inspection have gradually made it a bottleneck constraining product quality control. In recent years, automatic optical inspection (AOI) technology has gained widespread adoption in PCB surface defect detection due to its high efficiency and precision [17].

In the early stages of relevant research, many scholars integrated methods such as threshold segmentation [18], edge detection, and image segmentation [19] into AOI systems to achieve automatic detection of PCB surface defects. However, these traditional image processing approaches could only perform binary judgments on defect presence, failing to provide critical information such as defect categories and locations. Subsequently, traditional machine learning methods such as support vector machine [20], random forest [21], and Bayesian algorithm [22] were also integrated into AOI systems to achieve automatic classification of PCB surface defects. However, these traditional machine learning approaches heavily rely on feature engineering and struggle to accurately localize defects, resulting in significant fluctuations in detection accuracy. Deep learning methods employ an end-to-end feature learning mechanism that automatically extracts features from data, demonstrating significant advantages in the accuracy of PCB surface defect detection. Among these, the YOLO series models leverage their end-to-end detection capabilities to directly output target category and location information from input images, showing excellent performance in both detection accuracy and speed. Adibhatla et al. [23,24] employed YOLOv2 and YOLOv5 for binary classification detection of surface defects on PCBs, achieving detection accuracies of 98.82% and 99.74%, respectively. Zhao et al. [25] proposed a ShuffleNetV2-YOLOv5 model based on attention mechanisms and multi-source information fusion. Compared to lightweight models such as YOLOv3tiny and YOLOv4tiny, this model achieved higher accuracy with fewer floating-point operations. Tang et al. [26] developed a novel PCB-YOLO model architecture by improving YOLOv5, which optimized the regression process between prediction boxes and detection boxes using the EIoU loss function, significantly enhancing the model’s ability to locate tiny PCB defects. Yuan et al. [27] designed a novel YOLO-HMC model architecture for PCB defect detection by modifying YOLOv5. By eliminating the medium and large target detection heads and their corresponding feature pyramid structures in the original model, the proposed model achieved a favorable balance between detection accuracy and inference speed.

### 2.2. YOLOv8-Based Methods for PCB Surface Defect Detection

The YOLOv8 series models employ state-of-the-art backbone and neck architectures, focusing on maintaining an optimal balance between accuracy and speed while enhancing feature extraction and object detection performance. Consequently, these models have also been widely applied in industrial PCB defect detection scenarios. For instance, Khan et al. [28] evaluated the YOLOv8n and YOLOv8s models on the PCB surface defect dataset from the Peking University Open Lab on Human-Robot Interaction, with the mAP@0.5 of the two models reaching 95% and 98%, respectively. Yi et al. [29] introduced depthwise separable convolution into the backbone network and an efficient multi-scale attention module into the neck network of YOLOv8, further improving the model’s accuracy in detecting PCB defects. Liu et al. [30] proposed an improved YOLOv8-based PCB surface defect detection method, which adopted FasterNet as the backbone network to reduce unnecessary computational overhead and memory usage, and replaced the C2f module in the neck network with a C2f_Normalization-based attention module, further reducing the model’s computational load and parameter count. Li et al. [31] introduced the C2f-SimAM module into YOLOv8 and added a small target detection head, enhancing the model’s perceptual capability toward tiny PCB defects. Although existing YOLOv8-based PCB surface defect detection methods have achieved relatively satisfactory results, their performance in high-precision, real-time detection of multiple tiny defects under complex backgrounds remains to be further enhanced.

Compared with YOLOv8, Ultralytics’ newly released YOLOv11 achieves higher mean average precision and computational efficiency on the COCO dataset. However, it exhibits suboptimal performance in tasks involving limited data volumes and requiring fast transfer learning. Furthermore, YOLOv8 has been widely applied in surface defect detection across industries, including wood [32], steel [33], and insulators [34], and its comprehensive performance has been fully validated. Therefore, to facilitate the model’s deployment on resource-constrained edge computing terminals, this study selected the mature and stable YOLOv8 as the base architecture, and optimized its detection accuracy and computational efficiency.

## 3. Methods

### 3.1. Overall Framework of the GBE-YOLOv8 Model

**Figure 1:**
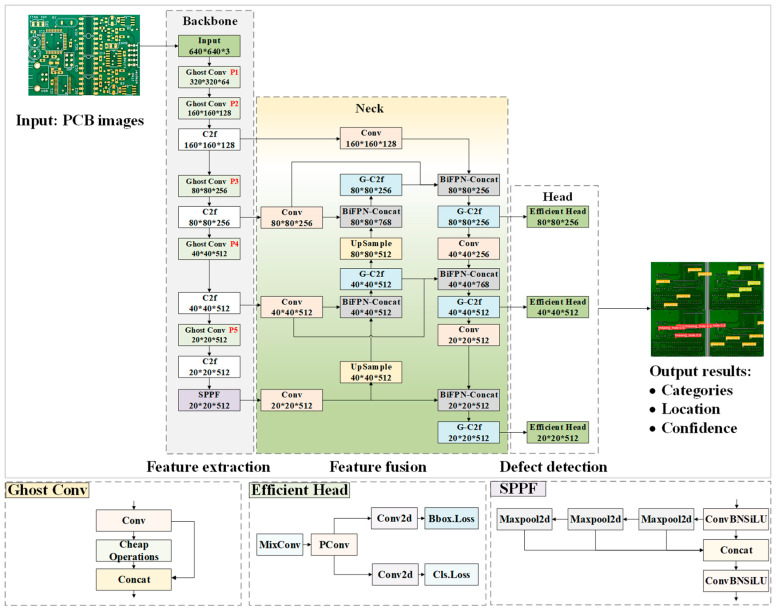
Model architecture of the GBE-YOLOv8.

This study designed a lightweight and high-precision GBE-YOLOv8 model for PCB surface defect detection, the architecture of which is illustrated in Figure 1. The GBE-YOLOv8 model comprises three core networks: a backbone network for feature extraction, a neck network for feature fusion, and a head network for defect detection. Specifically, the backbone network employs a lightweight Ghost Conv module to partially replace regular convolutions, effectively reducing the model’s computational load and parameter count while preserving the redundancy of feature maps, thereby improving detection speed. The neck network adopts a multi-stage feature fusion module named G-C2f and a dynamic weighting module named BiFPN-Concat to achieve deep fusion of defect features, enhancing the model’s ability to perceive information across different scales and semantic levels, thereby improving detection accuracy. The head network employs an Efficient Head that integrates mixed depthwise convolution and partial convolution, further optimizing the model’s detection accuracy and computational efficiency.

### 3.2. Improvement Methods of the GBE-YOLOv8 Model

#### 3.2.1. Ghost Conv Module

In YOLOv8n, redundancy in feature maps is primarily reflected in repeated activations in the spatial dimension and information overlap in the channel dimension, both of which compromise the model’s detection accuracy and inference speed. Directly discarding redundant feature maps would severely undermine the detection of tiny PCB defects. Therefore, this study selected to replace some regular convolutions in the backbone network of YOLOv8n with Ghost Conv module. This improvement effectively reduces the model’s computational load and parameter count while preserving the redundancy of feature maps, thus boosting its inference speed.

**Figure 2:**
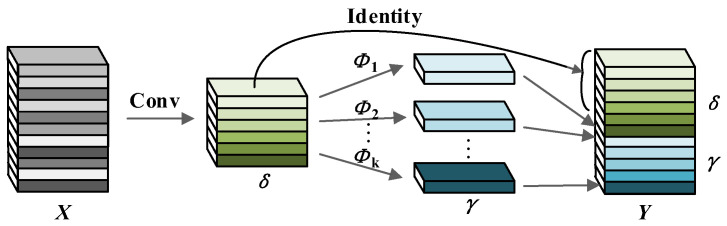
Network structure of the Ghost Conv module.

The network structure of the Ghost Conv module is illustrated in Figure 2. For a given input feature map *X*, regular convolution is first applied to generate intrinsic feature maps δ. Then, simple linear operations $\Phi_{i}$ are employed to map δ to ghost feature maps γ. Finally, δ and γ are concatenated to produce the complete output feature map *Y*. The calculation methods for δ and γ are presented in Equations (1) and (2), respectively.

(1) δ=X ∗ f

(2) $$\gamma_{ij}=\Phi_{i,j}(\delta_{i}),\;\;\;\;\;\;\forall i=1,2,3,\cdots ,m;j=1,2,3,\cdots ,s-1$$

In these equations, $X\in R^{h\;\times \;w\;\times \;c}$, where *h* and *w* denote the height and width of *X* respectively, and *c* denotes the number of channels of *X*; * denotes the convolution operation; *f* denotes the convolution kernel with $f\in R^{c\;\times \;k\;\times \;k\;\times \;m}$, where *k* × *k* represents the size of the convolution kernel and m denotes the number of intrinsic feature maps; *s* denotes the number of operations applied to each intrinsic feature map, which includes 1 identity mapping and s−1 linear operations; $\delta_{i}$ denotes the *i*-th intrinsic feature map; $\gamma_{ij}$ denotes the *j*-th ghost feature map derived from $\delta_{i}$; and $\Phi_{i,j}$ denotes the linear operation employed to generate $\gamma_{ij}$. Thus, the Ghost Conv module outputs a total of *m* × *s* feature maps. When generating output feature maps with the same size and channel count, the computational load and parameter count of Ghost Conv can be reduced to 1/*s* of those of regular convolution [35].

#### 3.2.2. G-C2f Module

PCB defects typically exhibit characteristics of complex backgrounds, small sizes, and subtle feature similarities. However, the C2f module in YOLOv8n struggles to effectively capture the subtle feature differences between various defects and its multi-scale fusion mechanism has limited adaptability to complex backgrounds, consequently leading to low model detection precision. Therefore, this study optimized the C2f module in the neck network of YOLOv8n into a G-C2f module. This improvement enhances the model’s ability to perceive information across different scales and semantic levels, specifically strengthening its ability to represent the features of small target defects, thereby improving the model’s detection precision for PCB defects.

**Figure 3:**
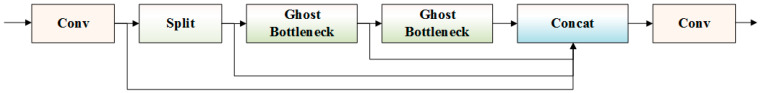
Network structure of the G-C2f module.

The network structure of the G-C2f module is illustrated in Figure 3. It adopts a multi-stage feature fusion architecture, with the processing flow divided into three key steps. First, input features undergo primary feature extraction via a convolution layer and the results are passed to the Concat module. Second, a split operation is applied to equally divide the feature map into two sub-feature maps with the same number of channels. One sub-feature map directly retains the original information and is transmitted to the Concat module, while the other undergoes deep feature enhancement through two cascaded Ghost Bottleneck modules. Finally, the Concat module performs concatenation and fusion of feature channels, followed by feature reorganization and dimensionality reduction through the output convolutional layer. By preserving parallel processing paths for both original and enhanced features, this design not only ensures the integrity of fundamental features but also enables in-depth mining of high-level features.

**Figure 4:**
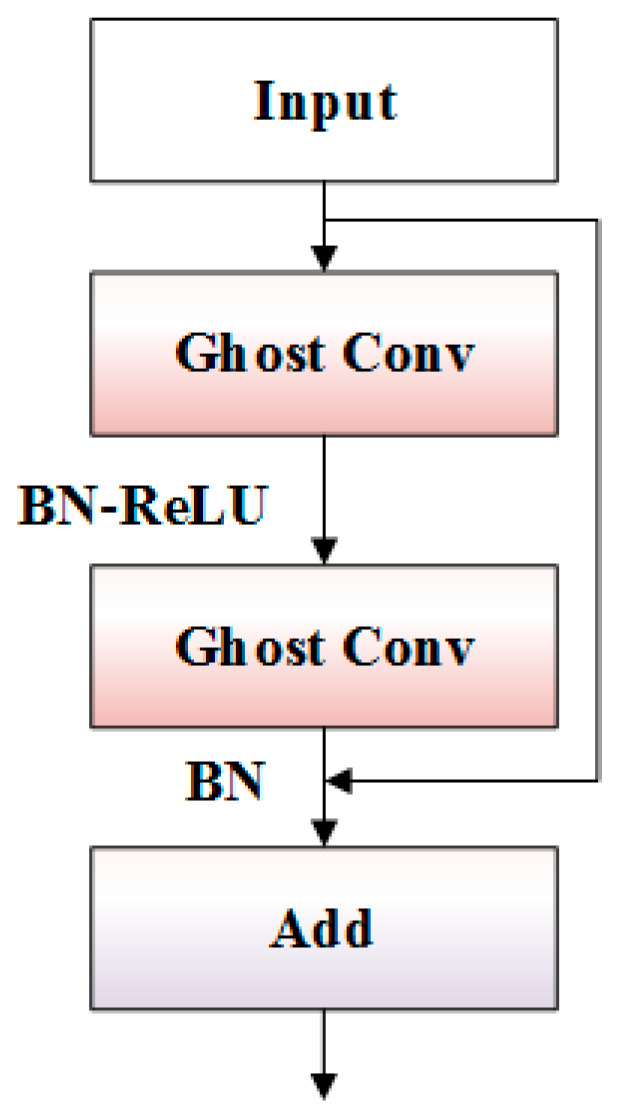
Network structure of the Ghost Bottleneck module.

The Ghost Bottleneck module, as a core component of G-C2f, significantly enhances computational efficiency by replacing regular convolutional operations with Ghost Conv. The network structure of the Ghost Bottleneck module is illustrated in Figure 4, and its processing flow is as follows. First, the input features undergo primary feature extraction through the first Ghost Conv layer, followed by feature normalization and nonlinear activation via the BN-ReLU combination. Subsequently, deep feature extraction is performed using the second Ghost Conv layer, with feature distribution adjustment achieved via BN. Finally, the original input features and the processed features are connected via a residual connection using the add operation.

#### 3.2.3. BiFPN-Concat Module

In PCB surface defect detection tasks, different categories of defects such as missing holes, short circuit, and spurious copper exhibit distinct multiscale characteristics in the feature space. The FPN + PAN module adopted by YOLOv8n employs an equalized feature fusion approach that fails to adequately account for the differential contributions of features at different scales to the final detection results. This leads to large-scale target features dominating the gradient backpropagation process, thereby suppressing the learning of small-scale target features and ultimately resulting in missed detections of tiny defects. To address this issue, this study referenced the design principle of BiFPN [36] and optimized the FPN + PAN module in the neck network of YOLOv8n into the BiFPN-Concat module. By dynamically assigning weighting coefficients to features of different scales, this module improves the model’s recall rate for tiny PCB defects.

**Figure 5:**
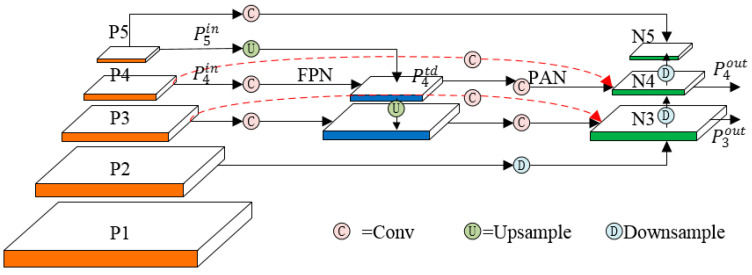
Network structure of the BiFPN-Concat module.

The network structure of the BiFPN-Concat module is illustrated in Figure 5. It employs fast normalized fusion to dynamically determine the weighting coefficients of features, and adaptively conducts weighted fusion based on the contribution of each feature map to the output. The calculation process of this fusion mechanism is presented in Equation (3).

(3) $$O=\sum_{i}\frac{w_{i}}{\varepsilon +\sum_{j}w_{j}}\cdot I_{i}$$

In the equation, $I_{i}$ denotes the input feature, $w_{i}$ and $w_{j}$ are learnable weights, O denotes the output feature, and ε=0.0001 is a small value introduced to avoid numerical instability. Meanwhile, the ReLU activation function is applied to $w_{i}$ to ensure $w_{i}\geq 0$. Taking the P4 layer in Figure 5 as an example, Equations (4) and (5) describe the feature fusion and output processes for this layer.

(4) $$P_{4}^{td}=Conv\left(\frac{w_{1}\cdot P_{4}^{in}+w_{2}\cdot ReSize(P_{5}^{in})}{w_{1}+w_{2}+\varepsilon }\right)$$

(5) $$P_{4}^{out}=Conv\left(\frac{{w_{1}^{’}\cdot P}_{4}^{in}+w_{2}^{’}{\cdot P}_{4}^{td}+w_{3}^{’}\cdot ReSize(P_{3}^{out})}{w_{1}^{’}+w_{2}^{’}+w_{3}^{’}+\varepsilon }\right)$$

In these equations, $P_{4}^{td}$ denotes the intermediate feature map of the fourth layer in the top-down path, $P_{4}^{out}$ denotes the output feature map of the fourth layer in the bottom-up path,Conv denotes the convolution operation, and ReSize denotes the upsampling or downsampling operation.

#### 3.2.4. Efficient Head Module

In PCB surface defect detection tasks, targets typically exhibit characteristics of complex backgrounds, small sizes, and similar structures. The detection head of YOLOv8n adopts a design architecture with fixed-size convolution kernels, which suffers from a single-scale receptive field during feature extraction and struggles to accurately distinguish tiny defects amid complex background noise. To address this issue, this study developed an Efficient Head that integrates mixed depthwise convolution (MixConv) [37] and partial convolution (PConv) [38] based on the decoupled dual-branch structure of the YOLOv8n detection head. This design enables the model to capture feature patterns of varying resolutions while reducing computational load.

**Figure 6:**
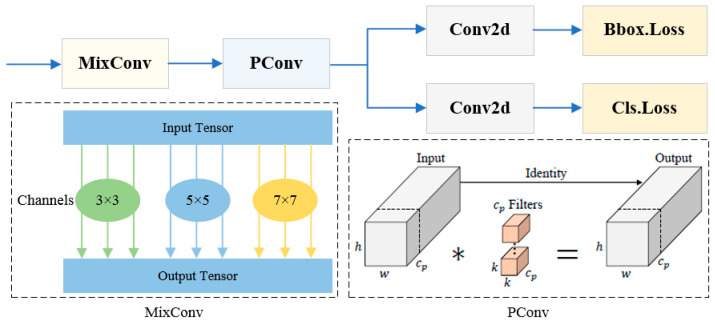
Network structure of the Efficient Head module.

The network structure of the Efficient Head module is illustrated in Figure 6. MixConv adopts the channel grouping strategy to divide the input feature map into several subspaces, and each subspace is processed by convolution kernels of different sizes. Finally, multi-scale feature fusion is realized by concatenation. In this study, the convolution kernels with three sizes (3 × 3, 5 × 5, and 7 × 7) were integrated in parallel to construct a MixConv module with a hierarchical receptive field structure. PConv is an efficient convolution design that conducts convolution operations only on a subset of input channels, while leaving the remaining channels unchanged. This sparse computation pattern can significantly reduce computational load. The FLOPs of regular convolution and PConv are computed according to Equations (6) and (7), respectively.

(6) $$F_{RConv}=h\times w\times k^{2}\times c^{2}$$

(7) $$F_{PConv}=h\times w\times k^{2}\times c_{p}^{2}$$

In these equations, h and w denote the height and width of the input feature map respectively, k denotes the convolution kernel size, and c denotes the number of input channels. For a typical partial ratio of $r=\frac{c_{p}}{c}=\frac{1}{4}$, the FLOPs of PConv are only 1/16 of those of regular convolution.

## 4. Experiments and Results

### 4.1. Experimental Design

#### 4.1.1. Dataset

**Figure 7:**
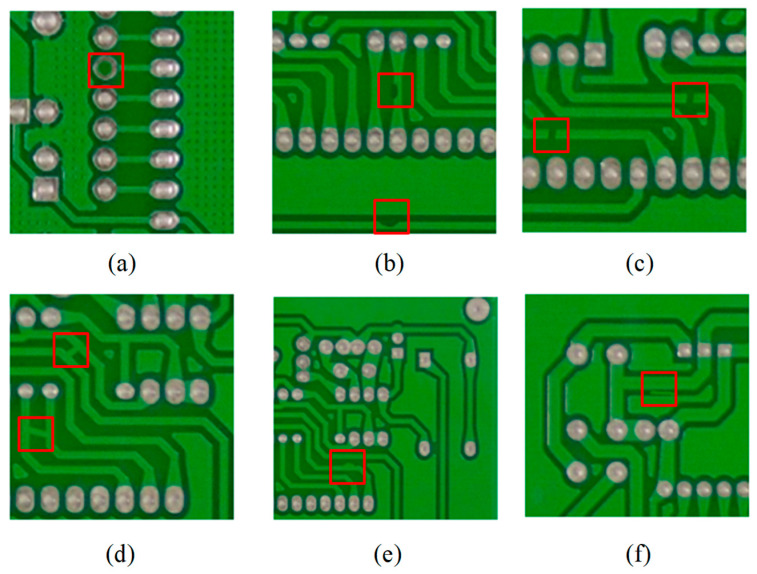
Examples of various types of PCB defects. The red box represents the location of the defect. (**a**) Missing_hole; (**b**) Mouse_bite; (**c**) Open_circuit; (**d**) Short; (**e**) Spur; (**f**) Spurious_copper.

This study adopted the publicly available HRIPCB dataset [39], released by Peking University, as the basic dataset for PCB defect detection. This dataset contains 693 images and covers six common types of PCB defects, namely missing_hole (Figure 7a), mouse_bite (Figure 7b), open_circuit (Figure 7c), short (Figure 7d), spur (Figure 7e), and spurious_copper (Figure 7f).

**Figure 8:**
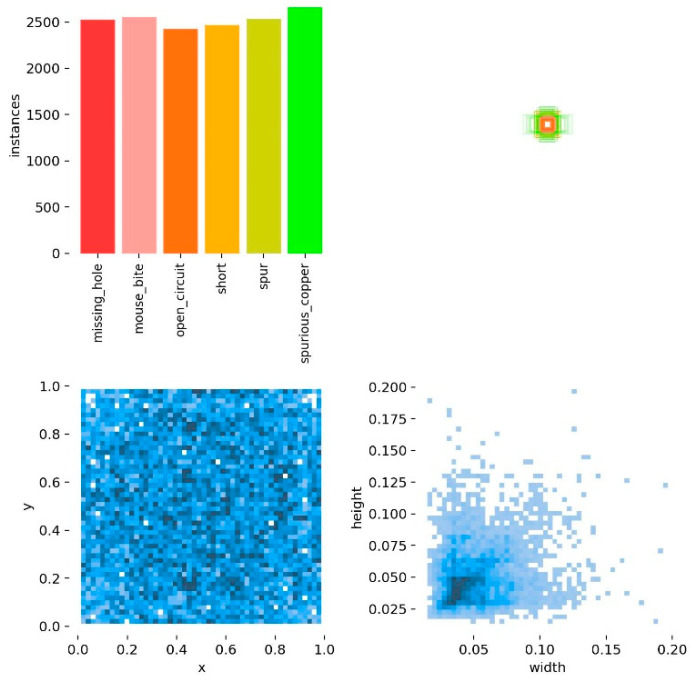
Label statistics for the HRIPCB-E dataset.

The six types of defect samples in HRIPCB dataset were randomly and evenly split into training set, validation set and test set according to the ratio of 7:2:1. Subsequently, given that the HRIPCB dataset has a limited number of samples, insufficient for fully verifying the model’s generalization ability, this study enhanced sample diversity of each subset through the application of image augmentation techniques, including geometric transformation, bounding box cropping, image filtering, and image noise injection. The post-augmentation dataset, named HRIPCB-E, contains a total of 10,668 images with the same size of 640 × 640 pixels. The label statistics for the HRIPCB-E dataset are illustrated in Figure 8. The number of samples for each defect type is relatively balanced, defect locations are evenly distributed across the images, the size of all defects does not exceed 4% of the total image size, and more than 60% defects are no larger than 0.25% of the total image size, classified as tiny defects.

#### 4.1.2. Experimental Environment and Model Hyperparameter Settings

This study conducted model training and testing on a workstation equipped with 15 vCPU Intel(R) Xeon(R) Platinum 8474C (Intel Corporation, Santa Clara, CA, USA) and NVIDIA GeForce RTX 4090D (NVIDIA Corporation, Santa Clara, CA, USA). For comparative experiments, the SSD, RT-DETR-L, YOLOv8n, YOLOv11n and GBE-YOLOv8 models were implemented using the PyTorch 2.0.1 deep learning framework. The hyperparameter settings for GBE-YOLOv8 are presented in Table 1. In the training phase, considering comprehensively the convergence speed, convergence stability, and convergence loss of the model on the validation set, the batch size was set to 16, the total number of epochs was set to 100, the initial learning rate was set to 0.01, the momentum parameter was set to 0.94, and the learning rate decay factor named gamma was set to 0.1. In the testing phase, the confidence threshold was set to 0.348 at which the model achieved the highest F1 score, the non-maximum suppression (NMS) threshold was set to 0.45 at which the model struck a good balance between avoiding duplicate detections and preventing missed detections, and all other parameters remained at their default values.

#### 4.1.3. Model Evaluation Metrics

This study adopted average precision (AP) and mean average precision (mAP) at intersection over union (IoU) thresholds of 0.5 and 0.5:0.95 as evaluation metrics for model detection accuracy, namely AP@0.5, AP@0.5:0.95, mAP@0.5, mAP@0.5:0.95. AP denotes the area under the precision-recall (P-R) curve for a given category. The horizontal axis of the P-R curve represents recall (R) and the vertical axis of the P-R curve represents precision (P). Both AP and mAP have a value range of [0,1], with larger values indicating better comprehensive detection accuracy of the model. Their calculation methods are presented in Equations (8) and (9), respectively.

(8) $$\mathrm{AP}=\int_{0}^{1}P\left(R\right)d\left(R\right)$$

(9) $$\mathrm{mAP}=\frac{1}{N}\sum_{i=1}^{N}{\mathrm{AP}}_{i}$$

In these equations, *P*(*R*) denotes the function of the P-R curve, *N* denotes the total number of sample categories, and AP*_i_* denotes the AP of the *i*-th category.

Meanwhile, this study also adopted giga floating point operations (GFLOPs) and the number of parameters as evaluation metrics for model complexity. GFLOPs represents the total number of floating-point operations the model requires during the inference phase, and is commonly used to evaluate the model’s computational complexity. Parameters represents the total number of parameters the model needs to learn during the training phase, and is a core metric for evaluating model lightweight performance. Smaller values of GFLOPs and parameters indicate lower model computational complexity and better lightweight performance. And frames per second (FPS) was used to evaluate the detection speed of the model.

### 4.2. Results and Analysis

**Figure 9:**
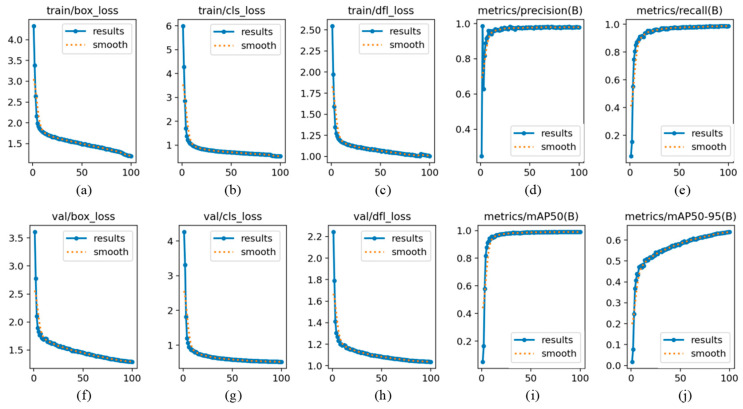
Iterative curves of model training and validation. (**a**) Changes in bounding box loss during training process. (**b**) Changes in classification loss during training process. (**c**) Changes in distribution focal loss during training process. (**d**) Changes in precision of bounding box during validation process. (**e**) Changes in recall of bounding box during validation process. (**f**) Changes in bounding box loss during validation process. (**g**) Changes in classification loss during validation process. (**h**) Changes in distribution focal loss during validation process. (**i**) Changes in mAP@0.5 of bounding box during validation process. (**j**) Changes in mAP@0.5:0.95 of bounding box during validation process.

The training and validation iteration curves of the GBE-YOLOv8 model on the HRIPCB-E dataset are illustrated in Figure 9. The model’s loss functions and evaluation metrics all converged within 100 epochs, exhibiting good stability and consistency. Thus, setting the total training epochs to 100 was validated as reasonable for this study. From the iteration process of the loss functions, both bounding box loss and classification loss of the model rapidly decreased to below 1.0, demonstrating the model’s strong capabilities in defect localization and classification. From the iteration process of the evaluation metrics, the model’s precision, recall, and mAP@0.5 all rapidly increased to over 0.9, and mAP@0.5:0.95 rose to over 0.6, further confirming the model’s high detection accuracy. Additionally, the smoothed curves of all loss functions and evaluation metrics show good consistency with their original curves, further indicating the model’s stable training process, with no significant overfitting or fluctuations observed.

The detection precision of the GBE-YOLOv8 model on six defect categories is presented in Table 2. Under the AP@0.5 metric, the model achieved detection precision exceeding 98% for all defect categories. Under the more stringent mAP@0.5:0.95 metric, the model also achieved detection precision exceeding 57% for all defect categories. For the larger defect type missing_hole, the model achieved maximum AP@0.5 and mAP@0.5:0.95 values of 99.4% and 64.0%, respectively. For the defect type short, which most closely resembles normal samples, the model achieved minimum AP@0.5 and mAP@0.5:0.95 values of 98.2% and 57.5%, respectively. These experimental results confirm that the model exhibits excellent recognition capabilities for various complex tiny PCB defects.

To further validate the advantages of the GBE-YOLOv8 model, this study conducted comparative experiments of its detection performance against advanced models including Faster R-CNN, RT-DETR-L, YOLOv8n, and YOLOv11n under identical experimental conditions. The comparative experimental results of each model are presented in Table 3. Among all compared models, the GBE-YOLOv8 achieved the highest mAP@0.5 of 98.9% and mAP@0.5:0.95 of 61.4%, and the smallest parameters of 2.6 M. Compared with the baseline YOLOv8n, the mAP@0.5, mAP@0.5:0.95 and FPS of GBE-YOLOv8 increased by 3.1%, 11.7% and 8.2%, respectively, while the parameters and GFLOPs of GBE-YOLOv8 decreased by 13.3% and 7.4%, respectively. Compared with the YOLOv11n, the mAP@0.5 and mAP@0.5:0.95 of GBE-YOLOv8 increased by 1.0% and 6.4%, respectively, while the parameters remained unchanged, the GFLOPs increased by 15.4%, the FPS decreased by 13.1%. To demonstrate the advantages of the proposed algorithm more comprehensively, and considering that many state-of-the-art baseline models are not open-source, we directly referenced experimental results from relevant studies for comparative analysis. Compared with the YOLO-MBBi [40], the mAP@0.5 and mAP@0.5:0.95 of GBE-YOLOv8 increased by 2.2% and 8.9%, respectively, while the parameters decreased by 70.5%. Compared with the GCC-YOLO [41], the mAP@0.5 and mAP@0.5:0.95 of GBE-YOLOv8 increased by 1.8% and 12.9%, respectively, while the parameters decreased by 68.3%. Compared with the Light-PDD [42], the mAP@0.5 and mAP@0.5:0.95 of GBE-YOLOv8 increased by 5.1% and 14.3%, respectively, while the parameters decreased by 32.3%. These experimental results demonstrate that the GBE-YOLOv8 achieves the highest detection precision while maintaining low model complexity, thus realizing the optimal balance between detection precision and model complexity.

**Figure 10:**
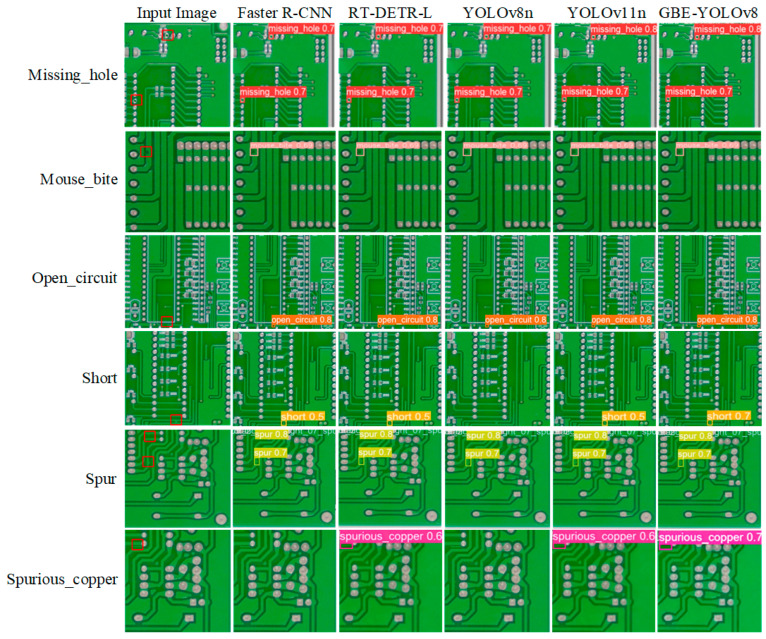
Visual comparative results of different models on six defect categories. The red box represents the location of the defect.

The visual comparative results of different models on six defect categories are illustrated in Figure 10, where each defect category is marked with a distinct color, and each label contains specific information about the defect’s location, category, and confidence score. These experimental results demonstrate that compared with other models, the GBE-YOLOv8 detects all defect categories with higher confidence scores and exhibits stronger reliability. Moreover, for the two defect types with the lowest detection precision in Table 2, namely short and spurious_copper, which are also the two most challenging defect types, the GBE-YOLOv8 shows the most significant improvement in the confidence scores. Specifically, for the short and spurious_copper defect types, the baseline YOLOv8n is more prone to missed detections, whereas the GBE-YOLOv8 proposed in this study can detect them more accurately.

**Figure 11:**
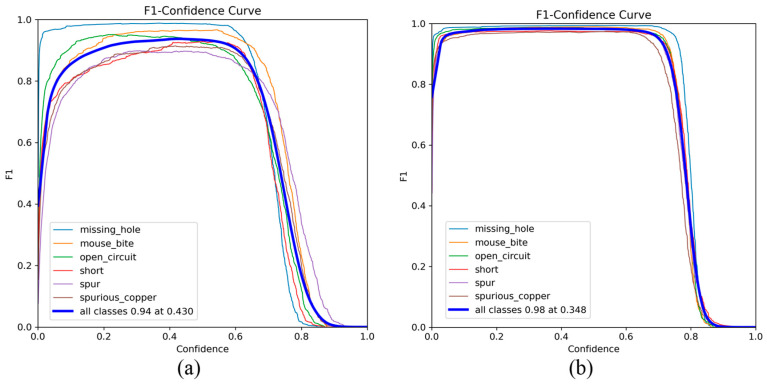
F1-Confidence curves of the YOLOv8n and GBE-YOLOv8. (**a**) YOLOv8n; (**b**) GBE-YOLOv8.

The F1-Confidence curves of the YOLOv8n and GBE-YOLOv8 are illustrated in Figure 11. The average F1 score of YOLOv8n reached the maximum value of 0.94 at the confidence threshold of 0.430, while the average F1 score of GBE-YOLOv8 reached the maximum value of 0.98 at the confidence threshold of 0.348. Compared with the baseline YOLOv8n, the F1-Confidence curves of GBE-YOLOv8 on six defect categories are smoother, with a higher and broader peak. Notably, the GBE-YOLOv8 shows the most significant improvement in F1 score for easily confused defect categories, such as spur and spurious_copper. These experimental results confirm that GBE-YOLOv8 achieves a superior balance between detection precision and recall, and exhibits stronger robustness in terms of confidence threshold adaptability.

To further validate the inference performance of the model in practical application scenarios, we selected an edge computing device named MIC-711-ON for deployment testing. This device is based on NVIDIA Jetson Orin Nano (CPU: 6-core Arm Cortex-A78AE v8.2 64-bit CPU; GPU: Up to 1024-core NVIDIA Ampere architecture GPU with 32 Tensor Cores; Memory: Up to 8 GB LPDDR5; NVIDIA Corporation, Santa Clara, CA, USA) and runs on the JetPack 6.0 operating system. During the experiment, the trained baseline YOLOv8n and the proposed GBE-YOLOv8 were first converted into the engine format. Subsequently, both models were deployed on the MIC-711-ON device to measure their inference speeds. The experimental results show that the FPS of YOLOv8n and GBE-YOLOv8 were 27 and 30, respectively, and the FPS of the improved model increased by 11.1%.

### 4.3. Ablation Studies

To thoroughly investigate the specific contributions of Ghost Conv, G-C2f, BiFPN-Concat, and Efficient Head to model performance improvements, this study conducted systematic module ablation experiments on the GBE-YOLOv8 and analyzed the function of each module using class activation heatmaps [43] to enhance the model’s interpretability. First, this study adopted YOLOv8n as the baseline model and introduced a lightweight Ghost Conv module into its backbone network to partially replace regular convolutions. Subsequently, G-C2f, BiFPN-Concat, and Efficient Head modules were introduced separately to analyze the performance contribution of each individual module. Finally, the above three improvement methods were introduced in pairs to evaluate the performance contribution of module combinations. The ablation experiment results of the GBE-YOLOv8 are presented in Table 4.

**Figure 12:**
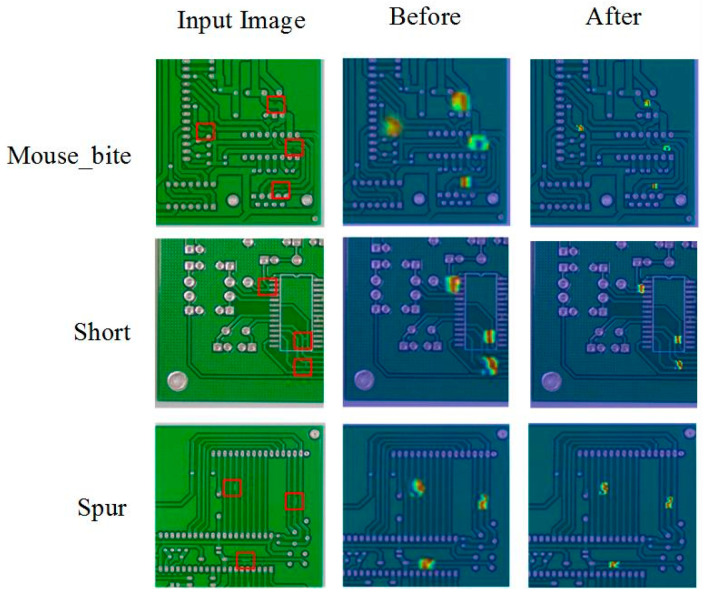
Class activation heatmaps before and after introducing the G-C2f module. The red box represents the location of the defect.

**Figure 13:**
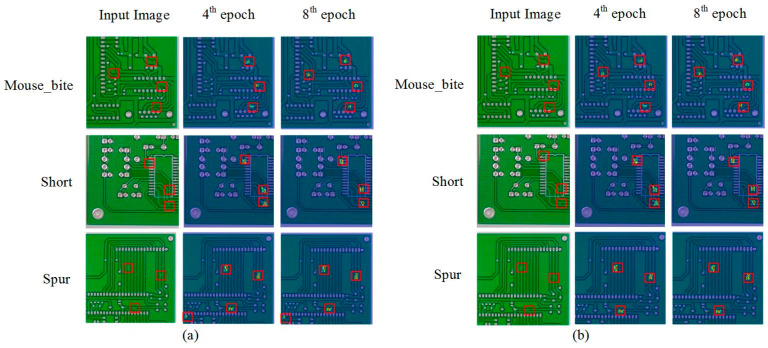
Class activation heatmaps before and after introducing the BiFPN-Concat module. The red box represents the location of the defect. (**a**) Without BiFPN-Concat module; (**b**) With BiFPN-Concat module.

**Figure 14:**
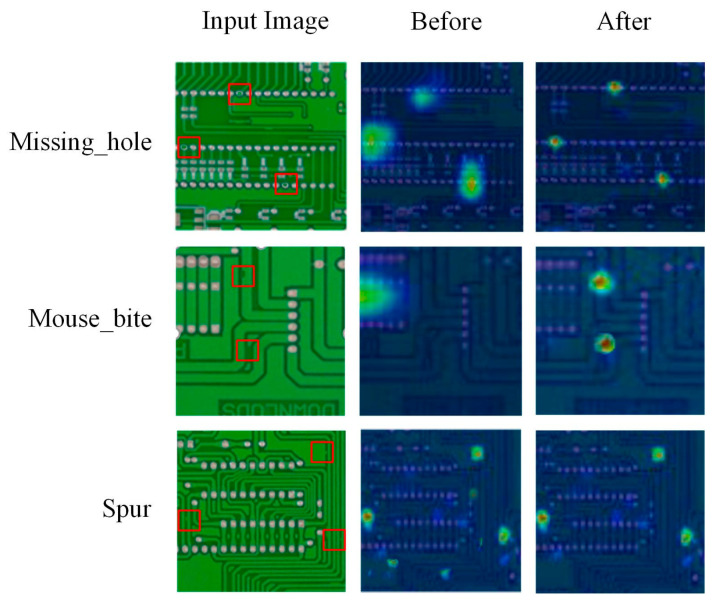
Class activation heatmaps before and after introducing the Efficient Head module. The red box represents the location of the defect.

(1) Ghost Conv: Compared with the YOLOv8n, by introducing the Ghost Conv module, the parameters and GFLOPs of the A model decreased by 16.7% and 8.6% respectively, while the mAP@0.5 and mAP@0.5:0.95 only slightly decreased by 0.2% and 0.7% respectively. These experimental results demonstrate that the Ghost Conv-based optimization can significantly reduce model complexity while maintaining high detection precision, thereby improving the model’s inference speed and laying a foundation for lightweight model deployment.
(2) G-C2f: Compared with the A model, by introducing the G-C2f module, the mAP@0.5 and mAP@0.5:0.95 of the AB model increased by 0.7% and 3.8% respectively, indicating that the G-C2f-based optimization can significantly improve the detection precision of the model. Comparing the AC and ABC models, as well as the AD and ABD models, it can be observed that the G-C2f module still enhances detection precision when using pairwise optimization methods. This indicates that the BiFPN-Concat or Efficient Head module does not suppress the contribution of the G-C2f module. Compared with the ACD model, the GBE-YOLOv8 achieved mAP@0.5 and mAP@0.5:0.95 improvements of 1.5% and 5.5% respectively, indicating that the combined application of all optimization methods can further enhance the contribution of the G-C2f module. The class activation heatmaps of the A model before and after introducing the G-C2f module are illustrated in Figure 12. The experimental results confirm that the G-C2f module can significantly suppress background noise while strengthening both the focus and intensity of the model’s response to various defect types.

(3) BiFPN-Concat: Compared with the A model, by introducing the BiFPN-Concat module, the mAP@0.5 and mAP@0.5:0.95 of the AC model increased by 0.8% and 3.9% respectively, indicating that the BiFPN-Concat-based optimization can significantly improve the detection precision of the model. Comparing the AB and ABC models, as well as the AD and ACD models, it can be observed that the BiFPN-Concat module still enhances detection precision when using pairwise optimization methods. This indicates that the G-C2f or Efficient Head module does not suppress the contribution of the BiFPN-Concat module. Compared with the ABD model, the GBE-YOLOv8 achieved mAP@0.5 and mAP@0.5:0.95 improvements of 1.4% and 5.2% respectively, indicating that the combined application of all optimization methods can further enhance the contribution of the BiFPN-Concat module. The class activation heatmaps of the A model before and after introducing the BiFPN-Concat module are illustrated in Figure 13. The experimental results indicate that before adding the BiFPN-Concat module, the model is prone to missed detection (mouse_bite) and false detection (spur) during the early stages of training. After adding the BiFPN-Concat module, the detection accuracy and convergence speed of the model can be significantly improved.

(4) Efficient Head: Compared with the A model, by introducing the Efficient Head module, the mAP@0.5 and mAP@0.5:0.95 of the AD model increased by 0.9% and 4.0% respectively, while the parameters remained unchanged and the GFLOPs only slightly increased by 1.4%, indicating that the Efficient Head-based optimization can significantly improve the detection precision while maintaining a low model complexity. Comparing the AB and ABD models, as well as the AC and ACD models, it can be observed that the Efficient Head module still enhances detection precision when using pairwise optimization methods. This indicates that the G-C2f or BiFPN-Concat module does not suppress the contribution of the Efficient Head module. Compared with the ABC model, the GBE-YOLOv8 achieved mAP@0.5 and mAP@0.5:0.95 improvements of 1.7% and 5.8% respectively, indicating that the combined application of all optimization methods can further enhance the contribution of the Efficient Head module. The class activation heatmaps of the A model before and after introducing the Efficient Head module are illustrated in Figure 14. The experimental results demonstrate that before adding the Efficient Head module, the model can accurately locate the defect when detecting the larger defect types such as missing_hole, but when detecting the smaller defect types such as mouse_bite and spur, the focus of the model is significantly deviated from the defect location, even resulting in missed detection and false detection. After adding the Efficient Head module, the localization accuracy of the model for various defect types has been greatly improved.

In summary, the use of each optimization method achieves the expected design purpose, and improves the performance of the model from different perspectives, such as reducing the number of parameters and computational load, and improving the detection accuracy. Furthermore, the combined application of G-C2f, BiFPN-Concat, and Efficient Head modules can further amplify the contribution of individual optimization method, enabling the model to strike the optimal balance between detection accuracy and complexity.

## 5. Conclusions

This study proposes a novel GBE-YOLOv8 model architecture for PCB defect detection based on the baseline YOLOv8n. Specifically, the backbone network of the model employs lightweight Ghost Conv to partially replace regular convolutions, thereby reducing the computational complexity and parameter count of the model. The neck network of the model utilizes the multi-stage feature fusion module named G-C2f and the dynamic weighting module named BiFPN-Concat to enhance the model’s feature representation capabilities for PCB defects. The head network of the model adopts an Efficient Head that integrates mixed depthwise convolution and partial convolution, further optimizing the detection accuracy and computational efficiency of the model. The experimental results demonstrate that compared with the baseline model and several other state-of-the-art object detection algorithms, the proposed GBE-YOLOv8 model exhibits significant advantages across key evaluation metrics, including detection accuracy, parameter count, computational complexity and detection speed. Additionally, the ablation studies confirm that each optimization module achieves its intended design goal and the combined application of all optimization modules enables the model to reach the optimal balance between detection accuracy and complexity.

Although the GBE-YOLOv8 model has achieved remarkable results in PCB defect detection tasks, there remains an issue worthy of further exploration. In this study, the model relies heavily on large quantities of labeled samples for training. However, in actual industrial scenarios, only a limited number of labeled PCB samples with various defect types are typically available. Therefore, it is necessary to further optimize the few-shot learning capability of the model using self-collected datasets from real-world scenarios.

## Figures and Tables

**Table 1:** Hyperparameter settings for GBE-YOLOv8.

Parameter Name	Parameter Value
Batch size	16
Epochs	100
Learning rate	0.01
Momentum	0.94
Gamma	0.1
Confidence threshold	0.348
NMS threshold	0.45

**Table 2:** Detection precision of the model on six defect categories.

Defect Category	AP@0.5 (%)	AP@0.5:0.95 (%)
Missing_hole	99.4	64.0
Mouse_bite	99.3	63.5
Open_circuit	98.7	59.0
Short	98.2	57.5
Spur	99.3	63.8
Spurious_copper	98.6	60.5

**Table 3:** Comparative experimental results of each model.

Model	mAP@0.5 (%)	mAP@0.5:0.95 (%)	Parameters (M)	GFLOPs	FPS
Faster R-CNN	92.7	45.0	34	150	13
RT-DETR-L	94.6	48.1	32	110	17
YOLOv8n	95.8	49.7	3.0	8.1	233
YOLOv11n	97.9	55.0	2.6	6.5	290
GBE-YOLOv8	98.9	61.4	2.6	7.5	252

**Table 4:** Ablation experiment results of the GBE-YOLOv8.

Model	Ghost Conv	G-C2f	BiFPN- Concat	Efficient Head	mAP@0.5 (%)	mAP@0.5:0.95 (%)	Parameters (M)	GFLOPs
YOLOv8n					95.8	49.7	3.0	8.1
A	√				95.6	49.0	2.5	7.4
AB	√	√			96.3	52.8	2.5	7.5
AC	√		√		96.4	52.9	2.6	7.6
AD	√			√	96.5	53.0	2.5	7.5
ABC	√	√	√		97.2	55.6	2.7	7.6
ABD	√	√		√	97.5	56.2	2.6	7.6
ACD	√		√	√	97.4	55.9	2.5	7.5
GBE-YOLOv8	√	√	√	√	98.9	61.4	2.6	7.5

Notes. “A” denotes YOLOv8n + Ghost Conv; “AB” denotes YOLOv8n + Ghost Conv + G-C2f; “AC” denotes YOLOv8n + Ghost Conv + BiFPN-Concat; “AD” denotes YOLOv8n + Ghost Conv + Efficient Head; “ABC” denotes YOLOv8n + Ghost Conv + G-C2f + BiFPN-Concat; “ABD” denotes YOLOv8n + Ghost Conv + G-C2f + Efficient Head; “ACD” denotes YOLOv8n + Ghost Conv + BiFPN-Concat + Efficient Head; “√” denotes inclusion.

## Data Availability

Publicly available datasets were used in this research. These data can be found here: https://robotics.pkusz.edu.cn/resources/dataset/ (accessed on 22 February 2026).
